# Ex-PRESS shunt implantation for intractable glaucoma with posterior chamber phakic intraocular lens: a case report

**DOI:** 10.1186/s12886-020-01784-4

**Published:** 2021-01-07

**Authors:** Rongrong Hu, Wei Xu, Baishuang Huang, Xiaoyu Wang

**Affiliations:** Department of Ophthalmology, The First Affiliated Hospital, Zhejiang University School of Medicine, Hangzhou, China

**Keywords:** Phakic intraocular lens, Implantable collamer lens, Glaucoma, Ex-PRESS shunt, Filtration surgery

## Abstract

**Background:**

Implantation of the posterior chamber phakic intraocular lens has been widely performed to correct high and extreme myopia. Chronic intraocular pressure (IOP) elevation may occur in its late postoperative period. For medically uncontrolled cases, surgical treatment is necessary, and benefits should be weighed against risks when determining whether to remove the lens.

**Case presentation:**

A 32-year-old man with extremely high myopia presented with progressive blurred vision and medically uncontrolled IOP in the right eye. His past ocular history was significant for bilateral implantable collamer lens (ICL) implantation ten years ago. On ophthalmic examination, the ICL was well placed with a vault height of 456 µm in the right eye. The anterior chamber angles were open but narrow, and mild to moderate trabecular pigmentation was noted. Ex-PRESS glaucoma filtration surgery without ICL removal was performed to control IOP. During surgery, an Ex-PRESS P50 shunt was inserted into the anterior chamber via the front edge of the blue-grey transition zone between the sclera and cornea. Transient hypotony and shallow anterior chamber occurred in the first week after surgery, along with an ICL tilt towards the cornea with reduced vault height. No other complications related to either the ICL or the Ex-PRESS shunt were noted. IOP remained stable at 12 ~ 14 mmHg at the first 3-month follow-up.

**Conclusions:**

Ex-PRESS glaucoma filtration surgery might be a safe and effective alternative treatment for intractable glaucoma with high myopia and ICL implantation. Careful assessment of the ICL position and anterior chamber angle is necessary to plan the appropriate surgical procedure. A postoperative shallow anterior chamber may result in ICL dislocation.

## Background

Posterior chamber phakic intraocular lens implantation is widely accepted and performed to correct high and extreme myopia. In this procedure, an implantable collamer lens (ICL) with a plate haptic design and anterior lens vaulting is injected through a clear corneal incision and placed in the posterior chamber and ciliary sulcus. Since its first implantation in 1993, multiple revisions have been made in material and design to increase applications and minimize complications [1]. Clinical investigations have demonstrated that ICL implantation provides satisfactory refractive outcomes and long-term stability [2–6]. Although relatively safe, ICL implantation is associated with complications, such as postoperative intraocular pressure (IOP) elevation, cataract formation, and corneal endothelial cell loss [7]. We present a case of open angle glaucoma with high myopia and a history of bilateral ICL implantation ten years ago. The IOP was not controlled well despite maximum tolerated anti-glaucoma medications, and Ex-PRESS glaucoma filtration surgery was subsequently performed.

## Case presentation

A 32-year-old man was referred for medically uncontrolled glaucoma. He had extremely high myopia and underwent bilateral ICL implantation surgery in 2009. Two months ago, he went to an outside hospital due to progressive blurred vision in the right eye and was diagnosed with glaucoma. He was treated with 4 topical anti-glaucoma medications, including Cartelol, Brimonidine, Latanoprost, and Brinzolamide; however, the IOP was not controlled well in the right eye.

**Fig. 1:**
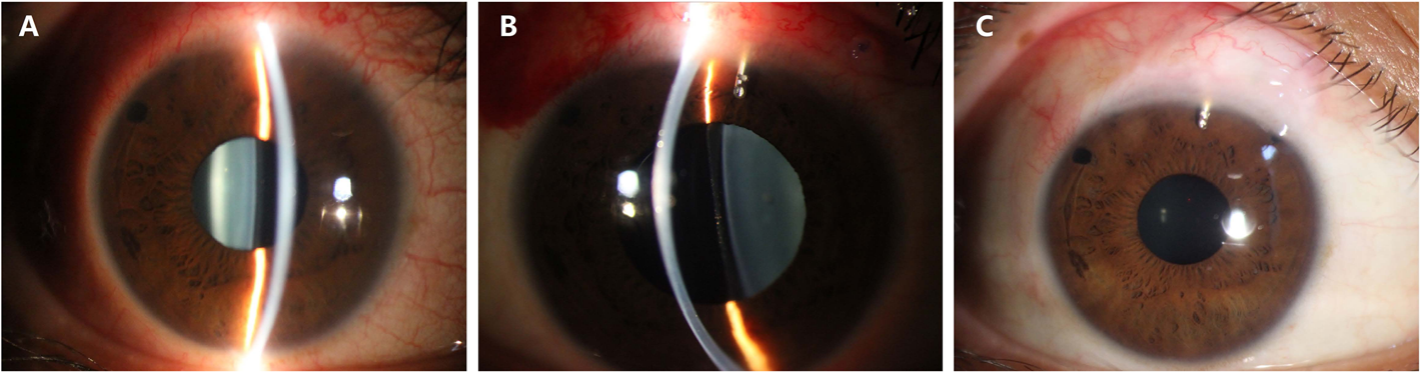
Slit-lamp photographs of the right eye. **a** The well-placed ICL and patent peripheral iridotomy on presentation. **b** The slightly shallow anterior chamber with the presence of ICL and Ex-PRESS shunt on postoperative day 1. The ICL appeared to tilt towards the cornea, and the pupil was dilated. **c** The well-placed Ex-PRESS shunt with a diffuse and avascular bleb 3 months after surgery

On presentation, the best corrected visual acuity (BCVA) was 20/50 in the right eye and 20/40 in the left eye. IOP was 37.3 mmHg in the right eye and 15.2 mmHg in the left eye (CT-80, Topcon Corp., Tokyo, Japan). On slit-lamp examination, the presence of well-placed ICLs without a central hole and patent peripheral iridotomies were noted in both eyes (Fig. 1a). The crystalline lenses were clear, and the fundus showed leopard change, Fuchs spots, lacquer cracks, and large parapapillary atrophy. The corneal endothelial cell density was 2569 cells/mm^2^ in the right eye and 2747 cells/mm^2^ in the left eye. An optical coherence tomography (OCT) scan revealed significant thinning of the mean retinal nerve fibre layer thickness in all sectors in both eyes. The standard automated perimetry (Humphrey Field Analyser, 30 − 2 pattern, Carl Zeiss Meditec, Dublin, CA, USA) revealed severe visual field loss with mean deviations of -23.25 dB and − 23.70 dB in the right and left eyes, respectively. The B ultrasonography scan revealed posterior vitreous detachment and significant posterior scleral staphyloma in both eyes. The axial length was 34.32 mm in the right eye and 33.43 mm in the left eye.

**Fig. 2:**
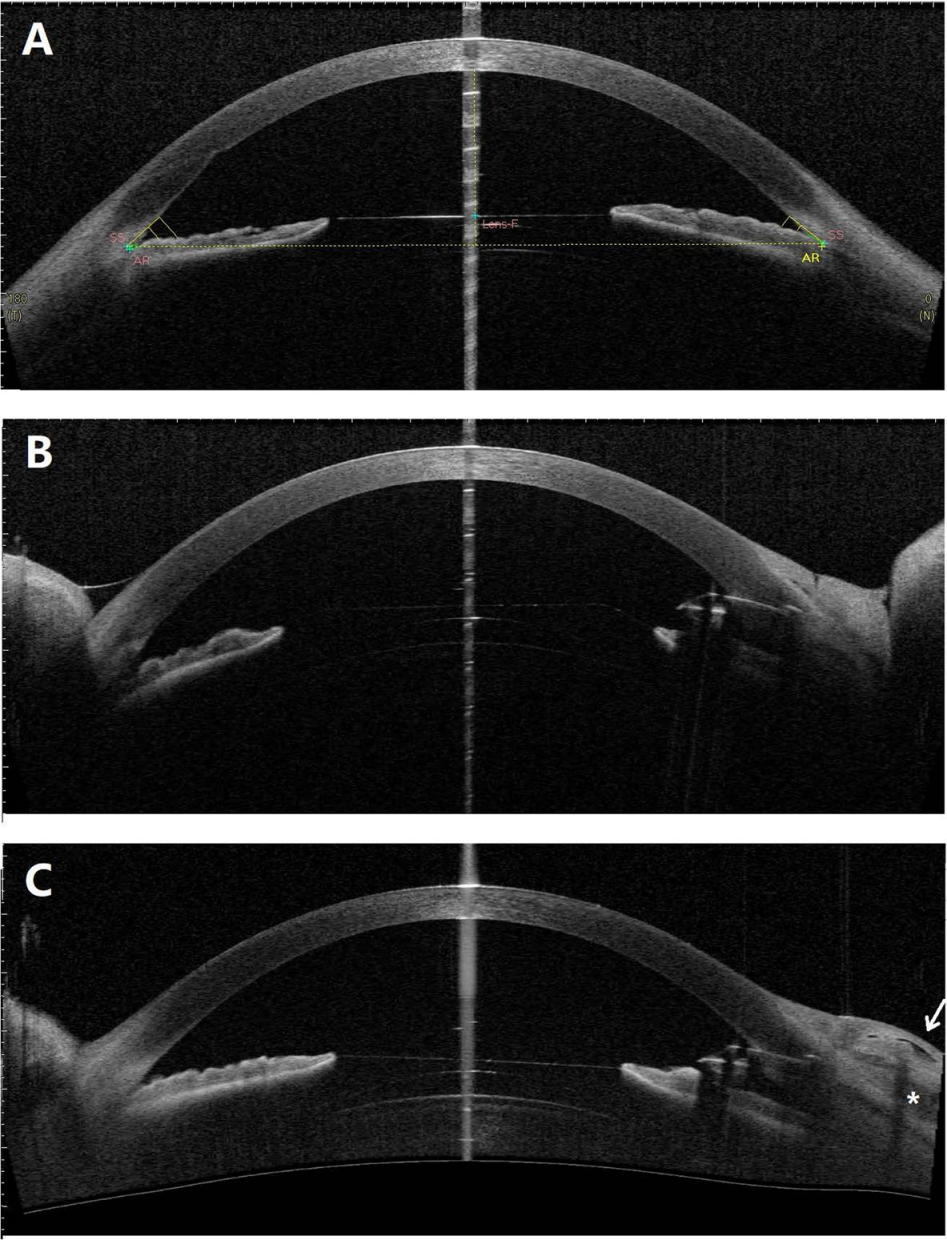
Anterior segment OCT images of the right eye. **a** The well-placed ICL and narrow trabecular-iris angles on presentation. **b** The slightly shallow anterior chamber with ICL tilting towards the cornea 2 days after Ex-PRESS shunt implantation. **c** The well-placed ICL and Ex-PRESS shunt with a well-functioning bleb 1 month after surgery. The internal fluid-filled cavity (white arrow) and hyporeflective area (*) are noted in the bleb

**Fig. 3:**
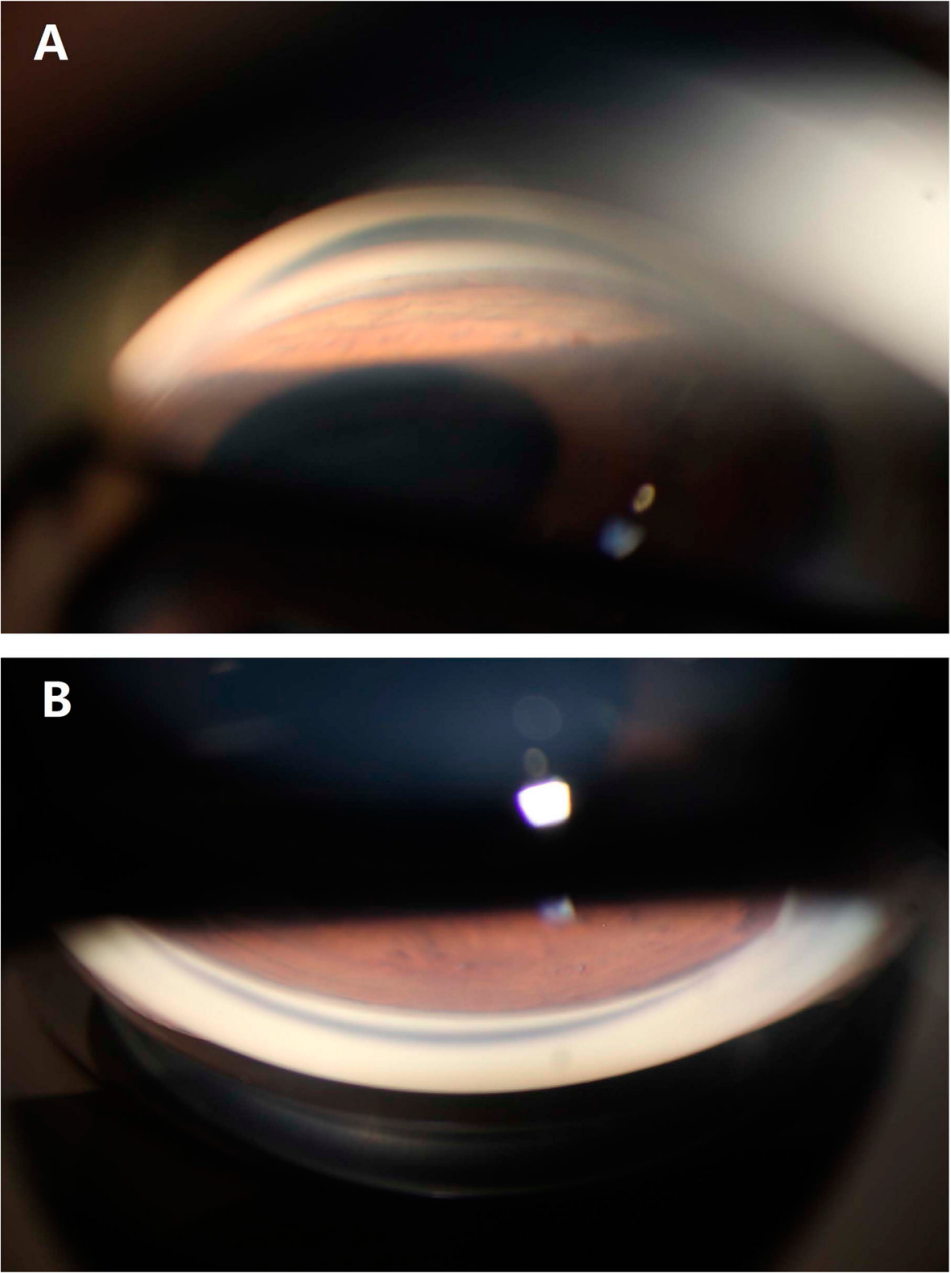
Gonioscopic photographs of the right eye. The angles were open but narrow (Shaffer grade II-III) with mild to moderate trabecular pigmentation in the inferior (**a**) and superior (**b**) quadrants

The ICL position and anterior chamber angles were carefully assessed with anterior segment OCT (CASIA, Tomey Corp., Nagoya, Japan). ICLs were well placed with vault heights of 456 µm and 629 µm in the right (Fig. 2a) and left eyes, respectively. The trabecular-iris angles at 500 µm from the scleral spur (TIA500) ranged from 13.5 degrees to 25.2 degrees in all quadrants of both eyes. The angle opening distance at 500 µm from the scleral spur (AOD500) was 0.27 mm and 0.22 mm in the superior quadrant of the right and left eyes, respectively. Gonioscopy showed that the angles were open but narrow (Shaffer grade II-III) with mild to moderate trabecular pigmentation in both eyes (Fig. 3a, b).

Ex-PRESS glaucoma filtration surgery was performed in the right eye. Under peribulbar anaesthesia, fornix-based peritomy and a 3*4 mm^2^ half-layer scleral flap were prepared in the nasal superior quadrant. A piece of cotton soaked with 0.4 mg/mL mitomycin-C was then placed under the scleral flap for 2 minutes before irrigation with balanced salt solution. With a 26-gauge needle, a penetration parallel to the iris plane was made into the anterior chamber at the front edge of the blue-grey transition zone between the sclera and the cornea, and viscoelastic agent was injected to maintain the peripheral anterior chamber. An Ex-PRESS P50 shunt (Alcon Laboratories, Fort Worth, TX, USA) was inserted into the anterior chamber via the penetration site. The scleral flap was sutured at the corners, and the conjunctiva flap was sutured closely. The patency of the shunt and the water-tightness of the bleb were examined by inflating the anterior chamber with balanced salt solution. The patient was treated with topical tobramycin-dexamethasone drops postoperatively, which was initially given per 2 hours for 3 days and then tapered weekly.

On postoperative day 1, the BCVA was 20/60, and the IOP was 6 mmHg. Slit lamp examination revealed a slightly shallow anterior chamber compared with the left eye (Fig. 1b). The ICL appeared to tilt towards the cornea and against the pupil margin (Figs. 1b and 2b). The ICL vault height was 431 µm based on measurements with anterior segment OCT on postoperative day 2. The IOP increased to 9 mmHg, and the anterior chamber depth increased after one week. One month after surgery, the BCVA was 20/50, and the IOP was 12 mmHg. The ICL returned to a well-placed position with a vault height of 451 µm (Fig. 2c). A functioning bleb with an internal fluid-filled cavity and hyporeflective area was noted by anterior segment OCT imaging. During the 3-month follow-up, the IOP remained stable at 12 ~ 14 mmHg, and the Ex-PRESS shunt was well positioned with a diffuse and avascular bleb (Fig. 1c).

## Discussion and conclusions

To the best of our knowledge, this is the first report of Ex-PRESS glaucoma filtration surgery in a highly myopic eye after ICL implantation without ICL removal. In the present case, the IOP was controlled well during the postoperative 3-month follow-up. Transient hypotony and shallow anterior chamber occurred at an early stage after surgery along with an ICL tilt towards the cornea with reduced vault height. No other complications related to either the ICL or the Ex-PRESS shunt were observed during follow-up.

IOP elevation following ICL implantation may occur in the early or late postoperative period due to various mechanisms. Remnants of viscoelastic material, steroid response, pupillary block, and overestimation of the ICL size were associated with early elevated IOP, which was typically observed within the first month postoperatively [4, 8–10]. With appropriate management, most cases were transient, and the IOP remained stable during follow-up. Chronic IOP elevation was less frequently reported in the literature, and its reported incidence ranges from 0–12.9% [2–6, 10–12]. Sánchez-Galeana et al. [13] reported a case of refractory IOP elevation with significant pigment dispersion following ICL implantation. A pigmented spindle on the cornea, increased pigment over the ICL, and dense pigment in the inferior angle and patent laser iridotomy were noted 6 months after surgery. IOP was uncontrollable with medical therapy, and ICL explantation and trabeculectomy were performed. Choi et al. [6] reported another case of pigmentary glaucoma after ICL surgery. In that case, glaucoma filtration surgery was performed 5 years after ICL implantation without ICL removal. In a 10-year follow-up of 133 eyes, Guber et al. [5] reported that 12 eyes (12.9%) developed chronic IOP elevation that required medical treatment, and pigmentation was observed in the angles in those cases. Pigment dispersion, which may result from chronic iris chafing, represents a likely cause for secondary glaucoma after ICL implantation in the long term.

In the present case, only mild to moderate trabecular pigmentation was observed in the anterior chamber angles rather than significant trabecular pigmentation. Hence, pigment dispersion alone may not be able to completely explain the mechanism of glaucoma. It is important to note that high myopia has been a well-established risk factor for glaucoma [14]. High myopia with myopic refractive error of greater than − 8 diopters was strongly associated with glaucoma [15, 16]. The myopic appearance of the optic nerve head frequently makes the detection of glaucomatous optic neuropathy difficult, and the diagnosis of glaucoma can be missed in highly myopic eyes when the IOP is within the normal range. We therefore hypothesise that the glaucoma was caused by mixed mechanisms in the present case. This case might be a missed case of juvenile open angle glaucoma with extremely high myopia that subsequently worsened following ICL implantation associated with pigment dispersion.

Due to the likely mixed mechanisms, the IOP may not be controlled well by ICL explantation alone. In the case of ICL explantation, clear lens extraction and intraocular lens implantation might then be required to correct the extremely high myopia for this 32-year-old patient who is in his working years. The intraoperative IOP fluctuations, which may exceed 70 mmHg while dropping close to 0 mmHg at times [17], would increase the extra risk of further visual field damage. Furthermore, with intraocular lens implantation, the patient would lose natural accommodation, which is preserved well in ICL refractive correction. For these reasons, we chose to retain the ICL and perform glaucoma filtration surgery for this young patient.

Ex-PRESS shunt implantation is equally efficacious as conventional trabeculectomy in reducing IOP with fewer intraoperative and postoperative complications [18, 19]. In this procedure, no tissue is removed, and the aqueous humour is diverted from the anterior chamber to the subconjunctival space through the fixed-size stainless-steel lumen. The P50 model differs from the P200 model with a 150-mm diameter inner bar across the lumen, which was designed to limit the fluid flow [20, 21]. In vitro experiments demonstrated that the effective luminal diameter was actually greater than 50 µm with the P50 model. As reported by meta-analysis, the Ex-Press shunt was associated with a significantly reduced frequency of postoperative hypotony and hyphema compared with trabeculectomy [22].

As there is no need for an iridectomy and no removal of scleral tissue, Ex-Press shunt would be less traumatic than trabeculectomy for an eye with ICL implantation. In the present case, we selected the Ex-PRESS P50 shunt to reduce the chance of intraoperative and postoperative changes in the anterior chamber depth, which may result in dislocation of the ICL. As observed in the first week after surgery, the ICL tilted towards the cornea with a reduced vault height along with anterior chamber shallowing and transient hypotony. Persistent postoperative hypotony and a severe shallow anterior chamber may cause irreversible changes in ICL position, or large mechanical contact between the ICL and the iris or between the ICL and the crystalline lens, resulting in chronic iritis, severe pigment dispersion, and cataract formation. Hence, great care should be taken to prevent persistent postoperative hypotony and shallow anterior chamber.

Ex-PRESS shunt implantation is typically not recommended for angle-closure glaucoma treatment due to the narrow peripheral anterior chamber. In myopic eyes with ICL implantation, the peripheral anterior chamber is commonly narrower than its natural status. We carefully assessed the peripheral anterior chamber parameters preoperatively. The AOD500 was 0.27 mm in the superior quadrant of the right eye, whereas the average external diameter of the Ex-PRESS P50 tube was 0.38 mm [21]. The peripheral anterior chamber would not be wide enough to accommodate the Ex-PRESS tube if the device was inserted through the blue-grey transition zone, which corresponds to the location of the trabecular meshwork. Hence, we chose the front edge of the blue-grey transition zone as the penetration site that would allow more space to accommodate the Ex-PRESS tube. Postoperative anterior segment OCT imaging confirmed the suitable Ex-PRESS position without contact with either the iris or the cornea (Fig. 2c). Over a long run, the peripheral anterior chamber may progressively narrow due to the age-related lens thickening (cataract formation). In that case, a laser iridoplasty around the shunt may help resolve the mechanical contact between the Ex-PRESS and the iris [23]. In cases with significant cataract formation, ICL explantation and cataract surgery would substantially deepen the peripheral anterior chamber.

Long-term follow-up of IOP is necessary after ICL implantation in highly myopic eyes. IOP elevation is caused by a variety of aetiologies. Careful assessment of ICL position and anterior chamber angles is important to understand the mechanism of postoperative elevated IOP and plan appropriate treatment. Our case demonstrates the feasibility of Ex-PRESS glaucoma filtration surgery for medically uncontrolled glaucoma without ICL removal. The postoperative shallow anterior chamber should be addressed with caution, as it may result in ICL dislocation. The long-term efficacy and safety of Ex-PRESS shunt implantation for highly myopic eyes after ICL implantation remains to be determined.

## Data Availability

The datasets used and/or analyzed during the current study are available from the corresponding author on reasonable request.

## Abbreviations

ICL: Implantable collamer lens
IOP: Intraocular pressure
BCVA: Best corrected visual acuity
OCT: Optical coherence tomography
TIA500: Trabecular-iris angles at 500 µm from the scleral spur
AOD500: Angle opening distance at 500 µm from the scleral spur
